# Circulating Antinuclear Antibodies in Patients with Pelvic Masses Are Associated with Malignancy and Decreased Survival

**DOI:** 10.1371/journal.pone.0030997

**Published:** 2012-02-17

**Authors:** Niels H. H. Heegaard, Mikkel West-Nørager, Julia T. Tanassi, Gunnar Houen, Lotte Nedergaard, Claus Høgdall, Estrid Høgdall

**Affiliations:** 1 Department of Clinical Biochemistry and Immunology, Statens Serum Institut, Copenhagen, Denmark; 2 Department of Pathology, Rigshospitalet, University of Copenhagen, Copenhagen, Denmark; 3 The Gynecological Clinic, The Juliane Marie Center, Rigshospitalet, University of Copenhagen, Copenhagen, Denmark; 4 Department of Pathology, Herlev Hospital, University of Copenhagen, Herlev, Denmark; University of Massachusetts Medical School, United States of America

## Abstract

**Background:**

Circulating autoantibodies occur more frequently in cancer patients than in patients without cancer.

**Methods and Findings:**

We examined sera from patients referred for pelvic mass symptoms to a tertiary university clinic. A total of 127 were diagnosed with epithelial ovarian cancer while 386 had a benign condition. A screen for IgG anti-nuclear antibodies (ANA) by indirect immunofluorescence on HEp-2 cells confirmed a highly significant overrepresentation of ANA in the cancer group where 40% had detectable (i.e., a titer ≥160) ANA compared with less than 12% in the benign group. The overrepresentation of ANA in the cancer group persisted (*p*<0.0001) after matching the age-profile of the benign group with the ovarian cancer group. Only 19 out of 127 patients in the age-matched benign subgroup were positive for ANA corresponding to an 85% specificity at 40% sensitivity of ANA as the only marker for malignancy. No correlation of ANA positivity in either group with specific bands in immunoblots could be demonstrated even though immunoblot positivity was clearly increased in the malignant group (41% vs. 3%). The presence, strength, and type of ANA did not correlate with serum CA-125 values or with staging, and ANA outcome did not contribute with independent diagnostic information. However, survival was significantly shorter in ANA-positive compared with ANA-negative cancer patients and patients with CA-125 below the median CA-125 value in the cancer group had a significantly decreased survival when positive for ANA. ANA status made no difference in the group with CA-125 values above the median. Also, there was a significant correlation between speckled ANA-strength and histological tumor grade.

**Conclusions:**

Circulating antibodies are a promising source for new biomarkers in cancer. Characterization of epitope specificities and measurements of consecutive samples will be important for further elucidating the role of ANA in evaluating ovarian cancer patients.

## Introduction

The immune system reacts to malignant tumor development by developing circulating antibodies against tumor proteins and specifically, since the body is tolerant to the normal proteins expressed in the body, by developing antibodies against cancer-associated post-translational modifications of these proteins as shown *e.g.* in ovarian cancer [1]. A rapid and amplified development of exquisitely discriminatory antibodies is a hallmark of the human immune system *e.g.* in infections and reactions against incompatible tissues in transplantation and transfusion. Despite the promises of arrays of recombinantly expressed proteins for detecting the reactivity patterns of cancer-specific antibody responses [2], studies using proteins purified from relevant tumor cell lines as antigens in screening for cancer specific antibodies show remarkably better results, *i.e.*, stronger reactivity and improved specificity [1]. When an immune response has evolved against a pathological cancer-associated epitope the reactivity may spread (epitope-spreading) to include epitopes present on parts of the normal protein structures because the proteins are being processed as foreign objects through recognition of altered parts of their structure. Such alterations may also be associated with various other pathologies *e.g.* in inflammatory and infectious conditions. This likely explains part of the reactivity of circulating antibodies in cancer sera against unmodified/processed recombinant antigens. The specific immunoreactivity against tumor antigens dependent on cancer-specific protein modifications seems a promising mechanism to exploit for developing more specific laboratory tests for cancer. In this way the immune system is a sensitive sensor of altered proteins in the body. The proteins themselves may or may not reach the circulation, and, if present only occur temporarily [3] and at very low concentrations and thus represent a challenge to measure. In contrast, once formed, the immune response is quite stably reflected by the circulating antibody population. The challenge in detecting the unique cancer-associated antibodies then is to choose the appropriate target antigens. The idea of using cancer cell line exosomes as antigenic targets for such quantitative immunoassays is attractive and has shown promising results in ovarian cancer studies as mentioned above. The antigen preparation for such assays is, however, complicated and difficult to standardize. Alternatively, it may be possible to use cancer cell lines optimized for other purposes as a tool to measure circulating antibody specificities supporting a cancer diagnosis. It has previously been shown in small studies that antinuclear antibodies (ANA) circulate more frequently in a number of different malignancies [4]. In this study we evaluate the HEp-2 cell line commonly used for screening for ANA associated with rheumatic disease to test for the presence of cancer-specific autoantibodies in sera from a large cohort of well-characterized patients referred to a tertiary university clinic for diagnostic work-up because of a pelvic mass. In total we examined the IgG response against HEp-2 cells in 558 patients of which 173 had some type of malignant ovarian conditions and the remainder had benign pelvic masses.

## Materials and Methods

### The Pelvic Mass Study

The Pelvic Mass study is a Danish prospective study of ovarian cancer, covering biochemistry and molecular biology with the purpose of identifying prognostic factors as well as factors that differentiate benign and malignant conditions. Samples originate from women referred to an outpatient clinical because of symptoms of a pelvic mass. The study was performed according to the Declaration of Helsinki including obtaining written informed consent from all participating patients. The study has been approved by the The Danish National Committee for Research Ethics, Capital Region (approval codes KF01-227/03 and KF01-143/04).

### Study Design

From September 2004 559 women admitted to the Gynecologic Clinic, Rigshospitalet, Denmark for surgery because of a pelvic mass, were enrolled. Of these patients 130 were diagnosed with ovarian cancer (127 epitelial, 3 non-epitelial), 26 with a borderline ovarian tumor, 386 with a benign disease, and 17 patients with non-ovarian cancer. All consecutive patients ≥18 years of age with the suspicion of a pelvic mass were informed both in writing and verbally and were invited after written consent to participate in the study. Patients were examined with an abdominal and vaginal ultrasound and serum CA-125 was analysed. Exclusion criteria were pregnancy, previous cancer or borderline tumor, no understanding of information or cancellation of planned surgery because of no suspicion of pelvic disease after further examinations.

All tissue specimens obtained during the surgery were examined by a pathologist specialized in gynecologic cancer. All patients are registered in Danish Gynaecologic Cancer Database (DGCD), which is a compulsory research and quality on-line database. The FIGO stage distribution for the ovarian cancer were 18 stage I patients, 12 stage II patients, 71 stage III patients and 26 stage IV patients. A total of 99 patients had serous adenocarcinama, 7 patients mucinous adenocarcinoma and 21 patients had tumors of other histological types.

For the borderline ovarian tumors the FIGO stage distribution were: 23 stage I patients, 2 stage III patients and one patient with a stage IV tumor. Of the 26 borderline ovarian tumors, 16 were serous and 10 were mucinous (Table 1).

**Table 1:** Study demographics in patients diagnosed with borderline ovarian tumor, ovarian cancer or a benign ovarian tumor.

	Ovarian cancer (N = 127)	Borderline ovarian tumor (N = 26)	Benign diseases (N = 386)	*p*-value
**FIGO stage**				
I	18	23		
II	12	0		
III	71	2		
IV	26	1		
**Tumor histology**				
Serous adenocarcinoma	99			
Mucinous adenocarcinoma	7			
Other*	21			
Serous borderline type		16		
Mucinous borderline type		10		
**Histological grade**				
Highly differentiated	17			
Moderately differentiated	55			
Poorly differentiated	50			
No grading	5			
**Status**				
Dead	73			
Alive	54			
**Age** (median/range)	66 (31–88)	55 (22–84)	45 (20–90)**	<0.0001
**CA125** (U/mL) (Median/range)	469 (6–12.310)	50 (12–853)	27 (2–1906)	<0.0001

*endometrioid adenocarcinoma N = 11, Clear cell neoplasms N = 6 and carcinosarcoma N = 4.

**No significant difference for the subset of 127 matched patients with benign conditions: Median age: 64 (range 54–90).

All ovarian cancer cases in this study were traced in the Danish Central Population Register (CPR) and date of death or emigration up to January 11th 2011, whichever came first, were registered. In addition, information about treatment (surgery and chemotherapy) and cause of death was established from the DGC database. During the follow-up period, a total of 73 OC patients had died from ovarian cancer (median follow-up time: 18 months, range: 1–68) and 54 patients were still alive (median follow-up time: 56 months, range: 38–75).

### Sample Processing

Blood samples were secured less than 15 days before surgery. All blood samples were collected and handled according to the conclusions of a previous study [5]. Handling and treatment of the blood samples were performed within one working day (*i.e.*, in less than 6 h). Thus, blood samples were left to clot at room temperature, and serum was isolated by centrifugation at 2000 g for 10 min. All sera were stored at −80°C in aliquots until analyses were performed.

### Laboratory Analyses

CA-125 values were determined in all sera by quantitative immunoassay (Brahms CA125 II Kryptor, BRAHMS GmbH) using ≥35 U/mL as a cut-off for positivity [6].

IgG ANA-screening on Hep-2 cells (ImmunoConcepts, Sacramento, CA) was performed by indirect immunofluorescence essentially as described [7]. HEp-2 originates from a human larynx carcinoma that was grown in rats [8] and is routinely used to detect and classify ANA that are characteristic for various autoimmune systemic connective tissue diseases [9]. It represents a standardized substrate for semiquantitative assessment of ANA reactivity (IgG antibodies) by indirect immunofluorescence. In brief, sera were diluted 1∶160 in PBS (phosphate-buffered saline) and 15–20 µl were applied to each well of the HEp-2 slides followed by incubation at room temperature for 30 min. After 10 min washing in PBS 15–20 µl fluorescein isothiocyanate (FITC)-labelled rabbit immunoglobulins against human IgG (diluted 1∶40 in PBS) were added and incubation continued for another 30 min. Wells were again rinsed for 10 min with PBS and the slides mounted in PBS with coverslips. Results were visually scored as negative (score 0), borderline positive (score 1), weakly positive (score 2), intermediate positive (score 3), or strongly positive (score 4). Positive samples were furthermore classified as cytoplasmic or nuclear and the latter was subdivided into 8 different antinuclear staining patterns by visual inspection of the fluorescence distribution.

Immunoblotting was performed using HeLa cell nuclear and cytoplasmic antigens (TriChem, Copenhagen, Denmark) that were separated by SDS-PAGE in 4–20% gels (InVitrogen, Copenhagen, Denmark) and transferred to nitrocellulose membranes (Whatman, Dassel, Germany). The membranes were blocked in TTN (50 mM Tris, pH 7.5, 1% Tween 20, 0.3 M NaCl) and individual lanes were inclubated with sera diluted 1∶100 in TTN for 1 h followed by 3× washing in TTN. The membranes were then incubated 1 h with alkaline phosphatase-conjugated rabbit immunoglobulins agains human IgG diluted 1∶1000 in TTN. After 3× washing in TTN, membranes were developed using BCIP/NBT substrate tablets (Kem-En-Tek, Copenhagen, Denmark).

### Data Handling

Unless otherwise noted only fluorescence scores of 2 and above were considered positive. The proportions in contingency tables were compared using Fisher's exact test and diagnostic performance was assessed using ROC curves. All graphical and statistical operations were performed using the Prism v. 5.0 program (GraphPad Software, San Diego, CA). Survival curves were estimated by the Kaplan-Meier method using log rank (Mantel-Cox) for statistical analysis of significance. The impact of IgG ANA (positive and negative) on mortality was estimated on the basis of the Cox proportional hazard model [10], applying the two-tiered scale using time since primary surgery as the time axis. The assumption of proportionality for the Cox model was assessed using Schoenfeld and martingale residuals as well as graphical methods using SAS, version 9.1. The regression analyses were adjusted for FIGO stage (I, II, III and IV), histological type of tumor (serous, mucinous, endometroid and other histological types), age at diagnosis (linear) and histological grade (high, moderate and low). Confidence intervals (95% CI) for the corresponding parameters in the multivariate COX regression model are presented. The analyses were performed using the SPSS 11.5 Statistical Software and graphs are constructed in the Prism program. Significance cut-off was *p*≤0.05.

## Results and Discussion

### Increased incidence of AntiNuclear Antibodies (ANA) in sera from patients with malignant pelvic masses

**Figure 1:**
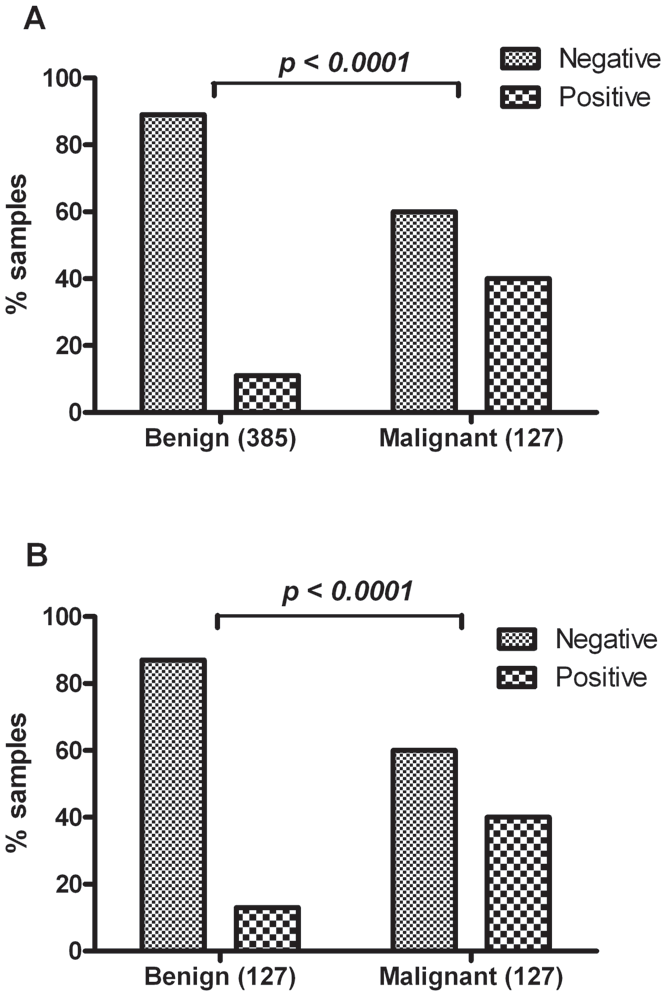
Proportion of positive results from the Antinuclear Antibodies (ANA)-screening of benign *vs.* malignant samples, Overall 40% of the patients with malignancies were positive for one or more ANA specificity (*cf.* also **Table 2**). This is a highly significantly fraction when compared to the patients with benign condition, both in the complete data set (A) and when comparing between age-matched groups (B).

All sera (Table 1) were tested for the presence of circulating IgG antibodies reacting with antigens in the HEp-2 cancer cell line used for screening of antinuclear autoantibodies (ANA). We find that circulating ANA are detected in patients with malignant pelvic conditions with a statistically highly significantly (p<0.0001) increased frequency compared with the controls, *i.e.*, patients suffering from benign pelvic conditions (Table 2). Patients with borderline conditions (n = 26) had ANA in 11% of the cases. Overall, 40% (51/127) of the patients with epithelial ovarian cancer were positive for antinuclear antibodies (Figure 1) compared with 11% in the group composed of women diagnosed with a benign ovarian tumor and with about 5% of the normal background population [11].

**Table 2:** The frequency of different Antinuclear Antibodies (ANA)-patterns in sera from patients with epithelial ovarian cancer and benign pelvic conditions.

	Positive, total	Centr.	Nucleoli	Mitotic Spindle	Nuclear Dots	Nuclear membr.	Homog.	Speckled	Cytopl.
**Cancer n (%) (n = 127)**	51 (40.2%)	2 (1.5%)	10 (7.9%)	3 (2.3%)	1 (0.8%)	0	5 (3.8%)	23 (17.4%)	12 (9.1%)
**Benign n (%) (n = 386)**	43 (11.2%)	1 (0.3%)	6 (1.6%)	0	2 (0.8%)	2 (0.8%)	13 (3.4%)	12 (3.1%)	9 (2.3%)
***p*** **-value**	<0.0001	NS	0.0013	0.0164	NS	NS	NS	<0.0001	0.0017

Fisher's exact test was used to obtain *p*-values. Some sera contain ANAs of more than one specificity. Centr., centromere; Nuclear membr., nuclear membrane; Homog., homogeneous; cytopl., cytoplasmic; NS, non significant.

There is no consensus [11] regarding the possibility that the frequency of ANA increases with age but several publications have suggested this to be the case [12], [13]. Since the median age of the group with benign conditions was significantly lower than the median age of the ovarian cancer cases (*cf.* Table 1) we did the analysis also in a subgroup of the benign cohort age-matched to the 127 patients with epithelial ovarian cancer (Figure 1B). Even though a slightly increased frequency of ANA was observed in the age-matched benign group (13%) this was still highly significantly lower (p<0.0001) than in the group with ovarian cancer. Thus in our patient population ANA were found about 4 times as often in ovarian cancer as in benign ovarian cases.

### Correlation of ANA with survival and tumor stage

**Figure 2:**
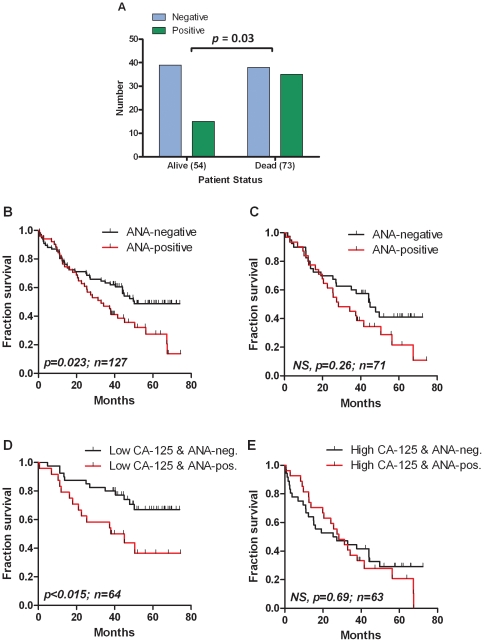
Mortality and presence of Antinuclear Antibodies (ANA). In (A) is shown the frequency of detection of ANA in the group of patients with epithelial ovarian cancer (n = 127) depending on their survival status (alive or dead). There is a statistically significant (p = 0.03) increase in the frequency of ANA in the group where the patients are dead. In (B–D) Kaplan-Meier survival curves are depicted. The graph in (B) indicates the survival of the 127 patients of whom 76 were negative for IgG ANA and 51 were positive. In (C) survival data grouped according to ANA status for the 71 patients with FIGO stage III cancer are shown. (D) shows survival of patients with CA-125 values below the median (469 U/mL) stratified according to ANA-status while (E) shows the same data for patients with CA-125 values above the median. NS, non-significant. Upward lines indicate instances of censored patients.

Overall, the presence of ANA was significantly correlated with survival, since 48% of the patients (n = 73) that died and only 28% of the surviving patients (n = 54) were positive for ANA (Figure 2A). This difference was statistically significant (p = 0.03). Conversely, there was no correlation between the incidence of ANA-positivity and tumor stage, *i.e.* the frequency of ANA in early (stage I–II) *versus* in late stage (stage III–IV) epithelial cancer was not statistically different. Also, no correlation were found between the histological type of tumor and the presence, strength, and pattern of ANA-staining.

Univariate Kaplan-Meier survival analysis was performed on all ovarian epithelial cancer patients (n = 127) and on FIGO stage III patients (n = 71) using the two-tiered scale (positive (all scores of 2 or above) or negative) cut-off for ANA (Figs. 2B & 2C). A significant difference between the two groups of patients were observed when all ovarian epithelial cancer patients were included (*p* = 0.023), whereas no significant difference were observed when stratifying stage III ovarian cancer patients (*p* = 0.124) (Fig. 2C).

In a multivariate Cox survival analysis of the 127 ovarian epithelial cancer cases, independent prognostic factors were found to be FIGO stage (P<0.0001: HR = 2.023, 95% CI: 1.417–2.889) and age at diagnosis (HR = 1.042, 95% CI: 1.020–1.066). No significant survival differences were seen among patients with the different levels of ANA (positive vs. negative) (*p* = 0.221), different histological types (*p* = 0.652) and histological grades (*p* = 0.427). The loss of significance in multivariate (Cox) analysis suggests that ANA is not an independent variable but associated with other known strong prognostic factors such as FIGO stage. Accordingly, while the presence of ANA is clearly associated with survival in univariate analysis of all samples (Fig. 2B) the significance is lost in the stage-III subset of samples (Fig. 2C).

In the group of 127 patients with epithelial ovarian cancer most (n = 122) had CA-125 values above the cut-off (35 U/mL). Analysis of survival in the two groups of patients with CA-125 values below or above the median value of 469 U/mL (n = 64 and 63, respectively) showed, as expected, a highly significant (p<0.0001) decreased survival in the high CA-125 group (data not shown). When adding ANA-status the data show that for patients below the median CA-125 valule (n = 64, of which 5 were below the cut-off of 35 U/mL) there is a statistically significant decreased survival if they are also positive for ANA (n = 24, p<0.015) (Fig. 2D). In patients with CA-125 values above the median (n = 63) there was no such difference in survival between the ANA-positive (n = 27) and ANA-negative (n = 36) patients (Fig. 2E).

### Correlation of ANA intensity and ANA patterns with malignancy and histological grade of tumor

**Figure 3:**
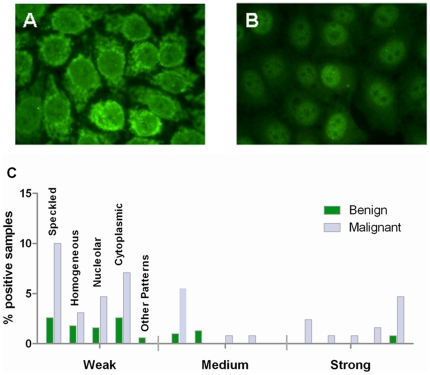
Antinuclear Antibodies (ANA) specificities. Examples of ANA-patterns of strongly positive sera staining in a cytoplasmic (A) and a speckled (B) pattern. In (C) is shown the distribution of the main types of ANA patterns as well as the signal strength in the benign and the malignant group. Strongly positive samples are almost only seen in the group with malignancy.

**Figure 4:**
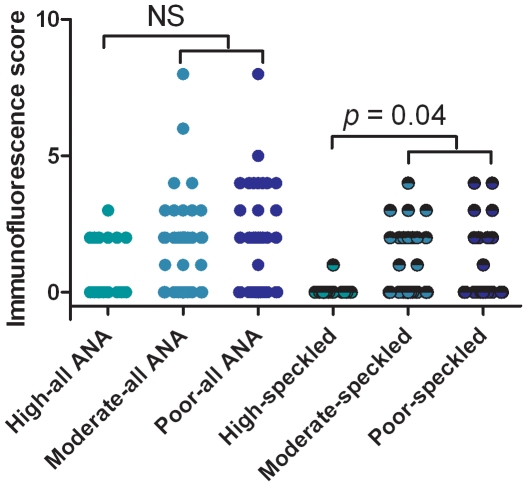
Relationship between ANA-staining intensity and histological grade of tumor (high, moderate, and poorly differentiated). While grading had no significant relationship with ANA-positivity of any specificity the presence of a speckled ANA pattern was significantly correlated with moderate-poor differentiation grade when compared with highly differentiated tumors (p = 0.04).

Routinely, immunofluorescence intensity is scored as negative or weakly, medium or strongly positive. Borderline positives were scored as negative in this study. Examples of strongly positive samples from patients with malignancies are shown in Figures 3A and 3B. It was found that, except in three benign samples, only samples from patients with ovarian cancer showed strongly reacting ANA (Figure 3C). Also, most of the ANA-positive samples belonged to one of four staining pattern groups: homogeneous, speckled, nucleolar, or cytoplasmic except for some strongly reacting samples with other patterns (mitotic spindle, nuclear dots and centromere patterns) in the malignant group. As seen by the numbers in Table 2 the speckled, nucleolar, and cytoplasmic patterns were statistically highly significantly more frequent in the samples from malignant cases while the incidence (3–4%) of ANA with a homogeneous staining pattern was the same in both groups and in fact is approaching the background frequency of this ANA pattern in healthy controls. A correlation of a speckled pattern with more aggressive disease was suggested by analysis of the histological grade of tumor. For the 127 samples from the group with epithelial carcinomas the histological grade was available from 122 while histological grade could not be typed in 5 cases (4 carcinosarcoma and one ovarian cancer case diagnosed by presence of cancer cells in the ascites fluid). Highly differentiated cancers were represented by 17 sera while moderately (n = 55) and poorly (n = 50) differentiated cancers represented the remaining samples in this group. We found that while ANA positivity occurred with the same frequency in the two groups (41% and 44% (high and low differentiation grade, respectively)) speckled patterns were practically absent (only one sample had a borderline positive pattern) in the highly differentiated group (Figure 4). This difference was statistically significant (p = 0.04).

### No predominant immunoblotting pattern

All available sera (375/385 benign and 167/179 malignant) were examined for IgG reactivity with specific protein bands on blots of SDS-PAGE separated HeLa-cell extracts. No predominant antigen specificity appeared and both unknown bands and a variety of bands with molecular weight characteristics of known antigens were observed (data not shown). A total of 41% of the epithelial cancer ANA-positive samples available for immunoblot analysis (18/44 available out of 51 ANA-positive) revealed bands (representing 7–8 different specificities) while only one out of the available ANA-negative malignant samples (74 available out of 76) was positive. For the entire benign group, a total of 11 samples (3%) (5 of which were ANA-positive) out of 375 (40 ANA-positive and 335 ANA-negative) available for testing were positive in immunoblotting and showed 4–5 different antigenic specificities.

### Performance of CA-125 as a diagnostic tool for ovarian malignancy with or without added ANA data

**Figure 5:**
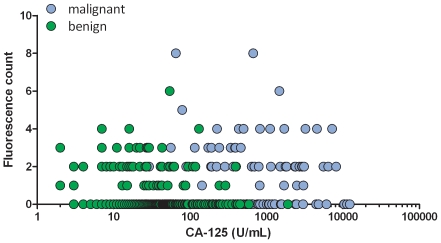
No correlation between serum-CA-125 values and the presence and intensity of ANA. The immunofluorescence score is depicted as a function of CA-125 values for the samples from benign (red symbols) and malignant cases (green symbols). Cut-off for positivity in the CA-125 test is 35 U/mL. All patients with benign ovarian tumors and epithelial ovarian cancers are included in this figure.

Apart from the prognostic information suggested by the significant findings in the survival curve analyses discussed above the presence of ANA did not appear to contribute overall with diagnostic information independently of serum CA-125. Using 35 U/mL CA-125 as the cut-off a sensitivity of 95% at 60% specificity was obtained from a ROC curve with an AUC of 0.93 for differentiating the benign from the group of patients with epithelial ovarian cancer (data not shown). Almost all the ANA-positive samples from malignant cases had serum CA-125 values above the cut-off (Fig. 5). Thus, including ANA-positivity as a parameter for the diagnosis of ovarian cancer only added one malignant sample which had a CA-125 value below 35 U/mL, while in the benign age-matched group we found 12 ANA-positive in the group with serum CA-125 below 35 U/mL and 7 in the group above 35 U/mL. Thus, for the age-matched sample set a serum that is double-positive (positive for ANA and a CA-125 value above cut-off) (n = 57) is 7 times more likely (7/254 vs. 50/254) to belong to the group of malignant samples, *i.e.*, 39% of the samples (50/127) from the malignant group and 6% (7/127) from the benign group are double-positive.

### Conclusions

The search for novel circulating biomarkers in ovarian cancer is intense and has harnessed a number of methods including proteomic analyses of the repertoire of circulating proteins and peptides [14]. However, despite much efforts no new biomarkers have yet emerged as an alternative to the established but imperfect CA-125 marker [6], [15]. Earlier work has shown that cancer-specific antigens may be released to the circulation in very low and transient concentrations. By virtue of being altered self-molecules, however, such antigens are likely to provoke an immune response. Accordingly, a number of studies have shown anti-cancer antibodies circulating in a wide variety of types of cancer patients [16]. In ovarian cancer this includes some recent studies where the immunoreactivity with specific proteins derived from cancer cell exosomes or cancer cell lines were clearly and specifically increased [1] for a number of antigen targets [17]. A more convenient way of testing for the presence of antigens present in cancer cells might be to use standardized cellular substrates from cultured cancer cells as in the present study.

Here, the disease sensitivity based on a positive ANA-result was 41% and while this is not a diagnostically useful sensitivity the numbers indicate that a positive ANA-screening is quite specific for malignancy in this group of patients, *i.e.*, only 42 out of 385 patients in the benign group had a positive result, corresponding to a specificity of about 89% in the group diagnosed with ovarian cancer. Using ANA as the only marker we got a comparable 85% specificity at 40% sensitivity in the age-matched samples sets. Moreover, in the group of patients with malignant disease the presence of ANA correlated with a worse prognosis. Notably, a homogeneous ANA was as frequent in the benign group as the malignant group while a speckled ANA-pattern specifically correlated with moderately to poorly differentiated tumors. There was no indication of a common protein specificity of these antibodies when tested on proteins extracted from HeLa cells.

Overall, the results of this study suggest that the demonstration of antinuclear antibodies may have some value as a prognostic biomarker in patients with epithelial ovarian cancer. In cases with CA-125 above cut-off but below the median value the presence of ANA indicated a subgroup of patients that had worse survival. This association was not observed, however, in the group of highly increased CA-125 values. These data indicate that the use of ANA may be focused on patients with intermediately increased CA-125 values but firm conclusions depend on further investigations in larger groups of patients. The data do not allow any conclusions to be drawn regarding changes in ANA levels during treatment. This will rely on studies with consecutive samples.

The mechanisms behind the association of ANA with worse prognosis are not clear but the magnitude of an immune response depends on the antigenic stimulus. Thus, for faster growing or larger tumors it would be expected that more immunogenic material stimulates the immune system. A future characterization of epitope specificities is an important part of elucidating the importance of antibodies with ANA specificities in the evaluation of ovarian cancer patients.
